# One spinal manipulation session reduces local pain sensitivity but does not affect postural stability in individuals with chronic low back pain: a randomised, placebo-controlled trial

**DOI:** 10.1186/s12998-024-00541-4

**Published:** 2024-05-31

**Authors:** João Paulo Freitas, Leticia Amaral Corrêa, Juliana Valentim Bittencourt, Karine Marcondes Armstrong, Ney Meziat-Filho, Leandro Alberto Calazans Nogueira

**Affiliations:** 1 Rehabilitation Science Postgraduation Program, Augusto Motta University Centre (UNISUAM), Avenida Paris, 84, Bonsucesso, Rio de Janeiro, RJ CEP 21041-020 Brasil; 2 Physiotherapy Department, Northern Parana State University (UENP), Paraná, Brazil; 3 Physiotherapy Department, Municipal Health Secretariat of Prudentópolis, Paraná, Brazil; 4 Physiotherapy Department, Federal Institute of Rio de Janeiro (IFRJ), Rio de Janeiro, Brazil

**Keywords:** Low back pain, Chronic pain, Postural balance, Musculoskeletal manipulation

## Abstract

**Background:**

Clinical practice guidelines recommend spinal manipulation for patients with low back pain. However, the effects of spinal manipulation have contradictory findings compared to placebo intervention. Therefore, this study investigated the immediate effects of lumbar spinal manipulation on pressure pain threshold (PPT) and postural stability in people with chronic low back pain (cLBP). Second, we investigated the immediate effect of lumbar spinal manipulation on pain intensity and the interference of the participant beliefs about which treatment was received in the PPT, postural stability, and pain intensity.

**Methods:**

A two-arm, randomised, placebo-controlled, double-blind trial was performed. Eighty participants with nonspecific cLPB and a minimum score of 3 on the Numeric Pain Rating Scale received one session of lumbar spinal manipulation (*n* = 40) or simulated lumbar spinal manipulation (*n* = 40). Primary outcomes were local and remote PPTs and postural stability. Secondary outcomes were pain intensity and participant’s perceived treatment allocation. Between-group mean differences and their 95% confidence intervals (CIs) estimated the treatment effect. One-way analysis of covariance (ANCOVA) was performed to assess whether beliefs about which treatment was received influenced the outcomes.

**Results:**

Participants had a mean (SD) age of 34.9 (10.5) years, and 50 (62.5%) were women. Right L5 [between-group mean difference = 0.55 (95%CI 0.19 to 0.90)], left L5 [between-group mean difference = 0.45 (95%CI 0.13 to 0.76)], right L1 [between-group mean difference = 0.41 (95%CI 0.05 to 0.78)], left L1 [between-group mean difference = 0.57 (95%CI 0.15 to 0.99)], left DT [between-group mean difference = 0.35 (95%CI 0.04 to 0.65)], and right LE [between-group mean difference = 0.34 (95%CI 0.08 to 0.60)] showed superior treatment effect in the spinal manipulation group than sham. Neither intervention altered postural stability. Self-reported pain intensity showed clinically significant decreases in both groups after the intervention. A higher proportion of participants in the spinal manipulation group achieved more than two points of pain relief (spinal manipulation = 90%; sham = 60%). The participants’ perceived treatment allocation did not affect the outcomes.

**Conclusion:**

One spinal manipulation session reduces lumbar pain sensitivity but does not affect postural stability compared to a sham session in individuals with cLPB. Self-reported pain intensity lowered in both groups and a higher proportion of participants in the spinal manipulation group reached clinically significant pain relief. The participant’s belief in receiving the manipulation did not appear to have influenced the outcomes since the adjusted model revealed similar findings.

## Background

Low back pain (LBP) is the primary cause of disability worldwide [1] despite the wide range of treatment options [2]. Nearly two-thirds of individuals with LBP will experience a new episode within one year [3]. Chronic low back pain (cLBP) has a notable association with social costs and impairment [2]. Despite the substantial burden of the LBP, the literature still does not identify one treatment as most appropriate. Non-pharmacological therapies represent the first-line recommendations for LBP care [4]. Many clinical practice guidelines recommend spinal manipulation for LBP care [5, 6]. Numerous healthcare professionals utilize spinal manipulation for pain relief and restoring functional performance [7]. Spinal manipulation leads to pain relief and improved function, similar to other recommended LBP therapies [8, 9]. Nonetheless, in comparison to sham manipulation or placebo intervention, the effect of spinal manipulation has contradictory findings [10, 11].

Simulated procedures may generate a placebo effect. Simulated interventions are frequently employed as controls in studies testing novel surgical methods or manipulations in individuals with pain [12]. The placebo effect is incredibly potent for subjective outcomes such as pain intensity [13], which leads to the idea that placebo responses in clinical trials could be reduced by using objective outcomes rather than patient-reporting instruments. Pressure pain threshold (PPT) is a semi-objective psychophysical measure of pain sensitivity, and postural stability as a measure of mechanical function may be relevant in assessing LBP. Spinal manipulation can influence pain perception. PPT is affected regionally by spinal manipulation in asymptomatic individuals [14] and patients with chronic low back pain [15]. The mechanism underpinning the improvement in the PPT may be related to a specific neurophysiological effect or following a clinical pain relief as a non-specific general effect [15]. However, the intervention-specific effects of PPTs in patients with LBP are not well-defined. Lumbar spinal manipulation had no specific response on PPT in previous studies with simulated interventions [16, 17], which could reflect the methodological limitations [e.g., underpowered between-group comparisons and the absence of an evaluation of the participant blinding [16], the inclusion of participants with no LBP at the time of the intervention [17, 18]]. Pain can affect the neuromuscular reactions necessary for an adequate balance, and patients with LBP exhibit reduced postural stability [19–22]. Therefore, PPT and postural stability may offer an objective way to compare the effects of spinal manipulation to those of a simulated intervention.

Growing amounts of data indicate that contextual factors like patient expectations and treatment beliefs significantly affect musculoskeletal pain [23]. Nonspecific effects appear to comprise nearly two-thirds of the overall effect of surgeries for many health conditions [24] and treatments for osteoarthritis pain [25]. Placebo therapies outperformed no interventions for pain relief in patients with cLBP in the short term [26]. Thus, a positive expectation could mask an intervention effect, but the extent of this effect remains uncertain. Patient expectations positively affect pain in both the short and long term and positively affect functional outcomes in the medium and long term in individuals with cLBP [27]. An investigation of the immediate effect of spinal manipulation against sham on PPT and postural stability of patients with cLBP could shed light on the specific elements of spinal manipulation. Moreover, assessing the patient’s perceptions about the treatment could reveal the influence of treatment expectation, which is one of the contextual factors.

This trial investigated the immediate effects of lumbar spinal manipulation on the PPT and postural stability compared with sham in people with nonspecific cLBP. Secondarily, we investigated the acute effect of lumbar spinal manipulation on the pain intensity in patients with nonspecific cLBP and whether participants’ perceptions of the treatment (active vs. sham) influenced the treatment effect on PPT, postural stability and pain intensity.

## Methods

### Design

A two-arm, parallel, randomised, placebo-controlled, double-blind superiority trial was conducted. This study was approved by a Research Ethics Committee, prospectively registered in the Brazilian Registry of Clinical Trials (REBEC) RBR-3ksq2c (WHO U1111-1252-3077), and reported following the checklist recommendations in Consolidated Standards of Reporting Trials (CONSORT) [28]. A protocol was published, including a detailed description of the methods and no protocol amendments were made during the conduction of the study [29]. Participants were randomly allocated at a 1:1 distribution to the experimental group (Group 1) receiving lumbar spinal manipulation technique or the sham group (Group 2) receiving a simulated lumbar spinal manipulation technique. Participants were informed that they would receive a spinal manipulation or a simulated spinal manipulation and that both techniques could produce therapeutic effects. They were referred to the physiotherapist responsible for the intervention, who performed spinal manipulation or simulation of spinal manipulation according to the group to which the participant was allocated. The participant and the examiner who performed the initial and final assessments were blinded to the group allocation.

### Participants, therapists, and centres

The trial was conducted at the Guairacá Integrated Clinics, Guairacá University Centre (UNIGUAIRACA), in Guarapuava, Paraná, Brazil. Participants were recruited by invitation established in an announcement in the Guairacá Integrated Clinics and advertisements through social networks. All the participants signed the informed consent form.

Participants were included in the study if they self-reported (1) nonspecific cLBP (lasting at least three months); (2) aged between 18 and 55 years; (3) with moderate or severe current pain intensity (at least 3 points on the Numeric Pain Rating Scale); (4) who were not undergoing physical therapy treatment for LBP; and (5) with no symptoms below the knee. We excluded participants with self-reported (1) chronic widespread pain; (2) ligament laxity or hyperflexibility; (3) pregnant women; (4) conditions that contraindicated the use of vertebral manipulation techniques at high speed and low amplitude (red flags) such as vertebral fractures, cauda equina syndrome, cancer, inflammatory rheumatic diseases, vertebral infections, bone tuberculosis; (5) any condition that could interfere with pain sensitivity measures (for instance, changes in skin sensitivity, neurological diseases, or psychiatric diseases); (6) any condition that could interfere with body balance (for instance, neurological diseases or vestibulopathy); and (7) score equal to or greater than 19 in the Brazilian version of painDETECT questionnaire [30]. After performing the pre-intervention evaluation, the examiner (Examiner 1) left the evaluation room to remain blind to the intervention, and a physiotherapist (Examiner 2) with experience in spinal manipulation entered the room to perform the manipulation technique or the simulated technique according to the randomisation. Examiner 2 also remained blinded to the outcome assessment. After the intervention, Examiner 2 left the room, and Examiner 1 returned to the evaluation room and repeated the same evaluation performed before the intervention.

### Randomisation

Participants were randomly allocated at a 1:1 distribution to the experimental group (Group 1), which received a lumbar spinal manipulation technique, or the sham group (Group 2), which received a simulated lumbar spinal manipulation technique. Participants were allocated using randomly permuted blocks of 4 and 6. Allocation was concealed sequentially and numbered consecutively (1 to 80) in sealed, opaque envelopes with an index card containing the group allocation. An independent examiner not involved in other phases assigned interventions. The same examiner opened the sealed envelopes after the informed consent form had been completed, and the participant carried out the initial assessment. The participants received a unique study enrolment number. In order to assess the success of blinding strategies, participants were asked what treatment they thought they received after the post-treatment assessment of PPT and postural stability by a research assistant who is not involved in other phases.

### Intervention

**Fig. 1:**
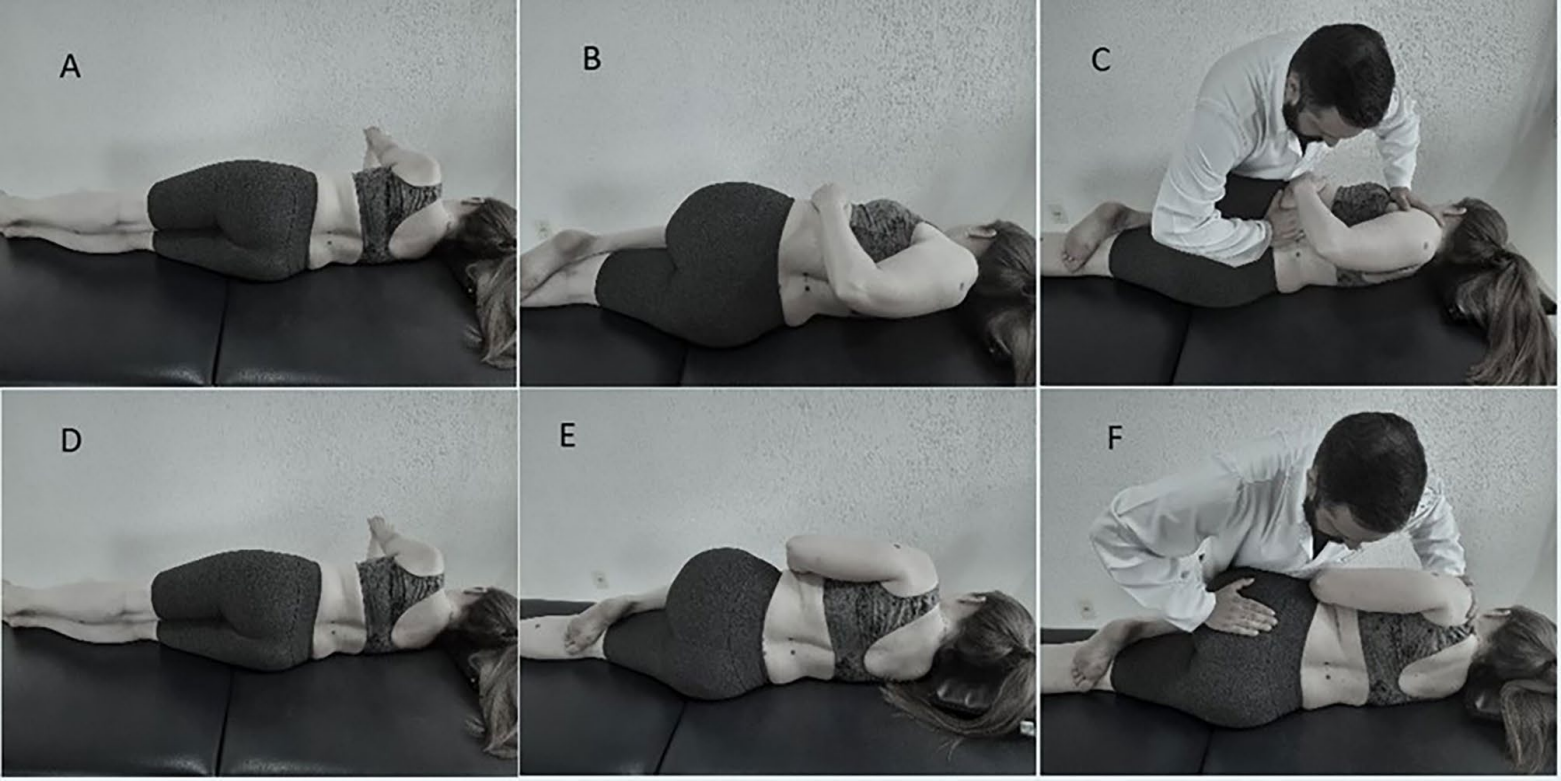
Spinal manipulation: Panel **A** - Initial positioning of the participant; Panel **B** - Final positioning of the participant; Panel **C** - Manual contacts and positioning of the physiotherapist. Simulated spinal manipulation: Panel **D** – Initial positioning of the participant; Panel **E** - Final positioning of the participant; Panel **F** - Manual contacts and positioning of the physiotherapist

Spinal manipulation was performed using the technique called *lumbar roll* by a physiotherapist with ten years of clinical experience. The participant was positioned in lateral decubitus with the target side up, knee flexed, and lower hip extended. The physiotherapist stabilised the participant’s shoulder with the physiotherapist’s cephalic hand and the participant’s thigh with the physiotherapist’s leg. Then, the physiotherapist made manual contact using the hypothenar region of the caudal hand over the transverse process on the upper side of the vertebra to be manipulated. The manipulation was performed with a passive rotation movement at high speed and low amplitude in the posteroanterior direction in association with the fall of the physiotherapist’s body [18, 31]. The manipulation was carried out bilaterally, starting from the symptomatic side. Treatment was considered complete in the presence of audible joint cavitation or after two attempts with no audible joint cavitation (Fig. 1).

The simulation of the spinal manipulation was based on current recommendations [11]. The simulated technique was performed similarly to actual manipulation by the same physiotherapist but with manual contact of the physiotherapist in the superior medial gluteal musculature in a broad and nonspecific way with the hand palm. The participants’ spine were kept neutral, and their hips were flexed 90°. The physiotherapist performed a slow, smooth, and unspecific impulse associated with a slight body fall, similar to a prior study [18]. The current technique was previously validated for placebo arms in spinal manipulation studies [32]. The simulated technique was carried out bilaterally twice, starting from the symptomatic side (Fig. 1).

### Outcome measures

The outcome measures were assessed as detailed in the published study protocol [29]. The primary outcomes were the PPT and postural stability immediately after the intervention. Secondary outcomes included pain intensity and the participant’s perceived treatment allocation immediately after the intervention.

#### Primary outcome

The PPT was measured using a digital algometer (model Force Ten FDX 25, Wagner Instruments, Greenwich, USA), recorded in kilogram-force (Kgf). A trained examiner evaluated the bilateral PPT before and after intervention using a digital pressure algometer with a 1 centimetre (cm)² rubber probe at six body sites: the mid-portion of the calf in the medial gastrocnemius muscle (MG), anterior tibial muscle laterally at the level of the anterior tibial tuberosity (AT), 2 cm laterally to the L5 spinous process (L5), 2 cm laterally to the L1 spinous process (L1), the mid-portion of the deltoid muscle (DT), and 2 cm distal to the lateral epicondyle (LE). The probe was placed perpendicular to the skin, and the pressure was increased at a rate of 500 g/second while the examiner visually monitored the force in real time by reading the digital display. The participant was instructed to say “stop” as soon as the pressure sensation became painful, so the examiner removed the algometer, and the threshold was recorded. Each body site was assessed three times, and the average of the three values was used. The PPTs are highly reliable when calculated as the mean of 3 measurements [33].

**Fig. 2:**
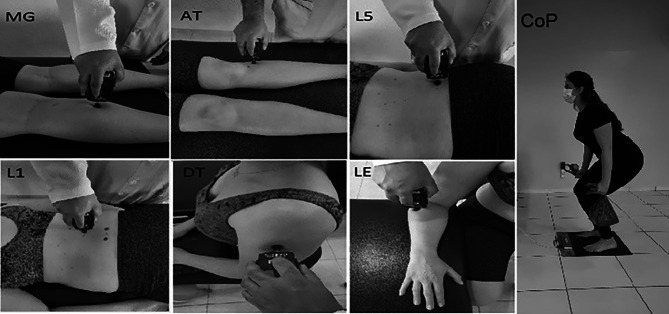
Primary outcome evaluation: body sites assessed by algometry (MG - medial gastrocnemius muscle; AT - anterior tibial muscle; L5 - L5 vertebra; L1 - L1 vertebra; DT - deltoid muscle; LE - lateral epicondyle) and evaluation of postural stability (centre of pressure)

Postural stability was evaluated via a baropodometric exam by displacing the centre of pressure (CoP) through the *FootWork* platform, with an active surface of 400 × 400 millimetres (mm). Participants stood barefoot on the platform, with their eyes opened and fixed on a mark placed two meters away. The participants were instructed to remain static in an anatomical position with their feet spaced at hip-width, with their elbows extended along the trunk, and holding in each hand a bag that weighed 2 kg in each hand. During the postural stability examination, the participant was instructed to perform as many squats as possible in 40 s. The stability variable investigated using the baropodometre was the area of ​​the CoP ellipse (A-CoP in cm^2^). The concurrent validity of the baropodometre in measuring the CoP displacement compared to the criterion measure of the force plate in patients with chronic non-specific low back pain was determined [34]. The bipedal static centre of pressure measures had acceptable intra- and inter-session reliability when assessed using a force plate [35]. Fig. 2 illustrates the primary outcomes evaluated.

#### Secondary outcome

*Secondary outcome*: Pain intensity was measured using the Numeric Pain Rating Scale (NPRS) from 0 (no pain) to 10 (worst possible pain) at the moment of evaluation. Pain intensity was assessed before and immediately after the intervention in both groups. A decrease in pain intensity assessed by the NPRS was considered clinically significant if a variation of at least 2 points between the pre-and post-intervention assessment was observed [36]. The influence of the contextual factor in the intervention was measured by a single question used to investigate the success of the blinding strategies. Participants answered what treatment they thought they received after treatment, with two response options: (1) spinal manipulation treatment or (2) sham spinal manipulation treatment. We planned to investigate participant’s expectations regarding the treatment using the following question: Thinking about how you felt before the treatment, how did you expect to feel after treatment? (1) worse, (2) a little worse, (3) neither better nor worse, (4) a little better, and (5) much better.

### Data analysis

The sample size calculation was performed a priori in the software G * Power version 3.1 (Heinrich-Heine-Universität, Düsseldorf, Germany) to determine a sufficient sample size. According to a model described previously to detect a minimum difference of 15% (effect size of 0.64) in the lumbar PPT, the statistical power of 80%, and an alpha of 0.05, the estimated sample size was 40 participants per group [37, 38]. A total of 80 participants were included in the present study.

The results were tabulated in a customised spreadsheet and analysed by an independent researcher blind to group allocation. The statistical analysis was conducted on an intention-to-treat basis. The results of the descriptive analysis are presented as the mean and standard deviation (SD) for continuous variables and as absolute values ​​and proportions (%) for categorical variables. The Shapiro-Wilk test revealed an approximately normal distribution of the primary outcomes. Levene’s test indicated no significant deviation from the variance homogeneity for all variables except for PPT at the left medial gastrocnemius muscle. One outlier in six variables (PPT at the left medial gastrocnemius muscle, PPT at the right L5, PPT at the right deltoid muscle, PPT at the left deltoid muscle, centre of pressure) was detected using the ROUT method with Q = 1.0%. Separate analysis without the outliers did not change the inferences. PPT, postural stability, and pain intensity were analysed using absolute values of change from baseline. Between-group mean differences and their 95% confidence intervals (CI) estimated the treatment effect. One-way analysis of variance (ANOVA) compared the differences between groups for each variable. ANOVA was conducted separately for each PPT location. One-way analysis of covariance (ANCOVA) was performed to assess whether beliefs about which treatment was received influenced the outcomes. Age and body mass index were also included as covariates due to the unbalanced distribution between groups despite randomisation. We performed one ANCOVA for each outcome variable (one analysis for each PPT, postural stability, and pain intensity). Variance inflation factors revealed an absence of multicollinearity among covariates since the values were below 1.2. We estimated percentages of change from baseline adjusted for baseline values for ease of interpretation. The percentage change from baseline was calculated using the following equation: (FV-IV/IV)*100, where “IV” represents the initial value of the given outcome and “FV” is the final value. Fisher`s exact test was used to calculate the significance of the difference between the groups regarding the number of participants who experienced clinically significant differences in clinical pain intensity. All the statistical tests were two-tailed with the pre-established significance level at *p* < 0.05. All data were analysed using the JASP version 0.14.1 software and GraphPad Prism software (GraphPad Software, San Diego, CA, USA) version 8.00 for MacBook.

## Results

**Fig. 3:**
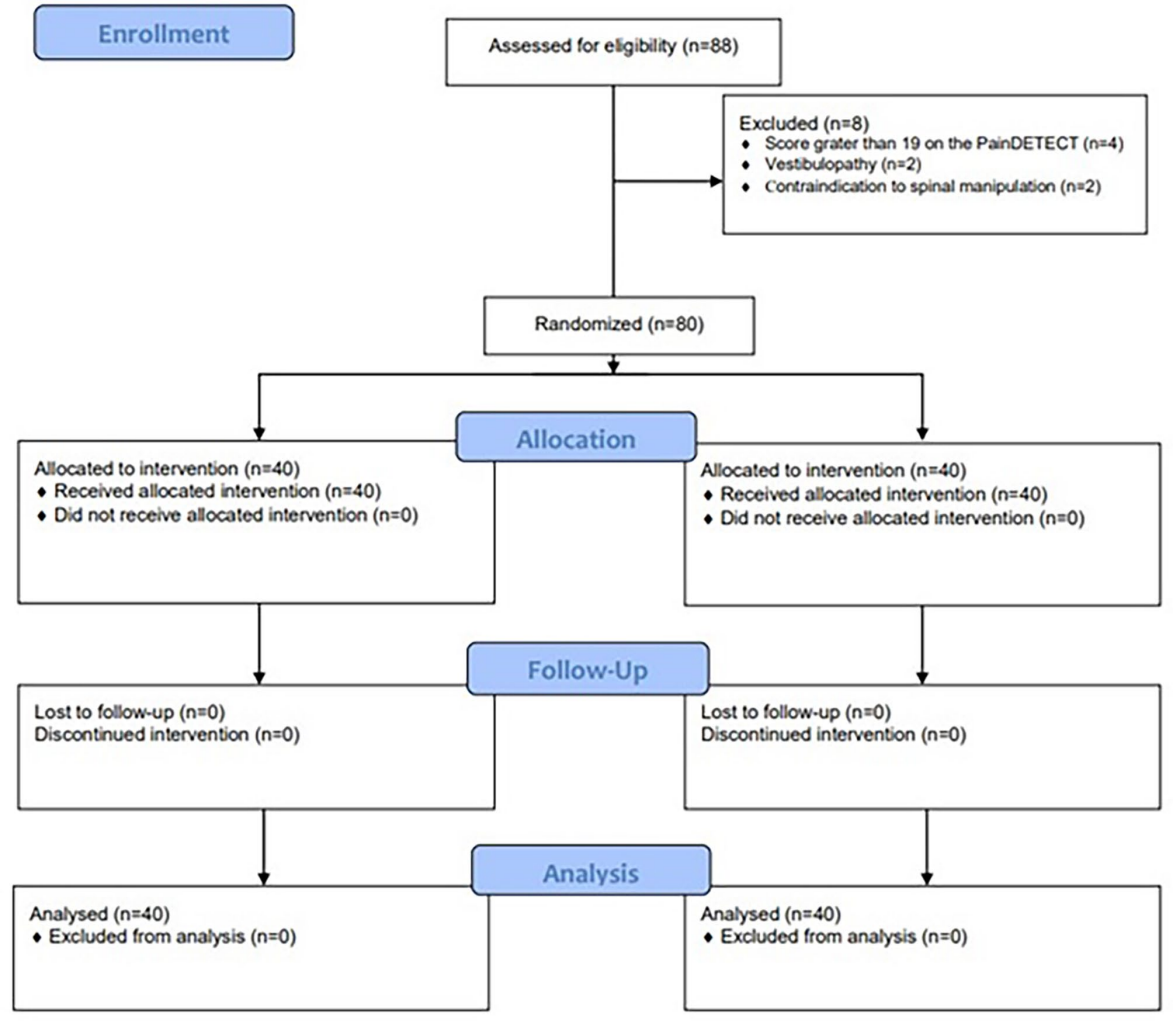
CONSORT flow diagram

A total of 80 participants with a mean age of 35 years (SD 11), of which 50 (63%) women who fulfilled the eligibility criteria were randomised equally to each group. Eighty-eight participants were screened from November 2021 to March 2022, and 8 were excluded. Fig. 3 presents a flowchart showing the number of eligible participants excluded and the reason for their exclusion. All participants completed the study and provided data on all outcome measures.

The groups were similar at baseline, except for age and body mass index. The sham group participants were older and had a higher body mass index than the participants allocated to the spinal manipulation (Table 1).

**Table 1:** Participant baseline characteristics

	Spinal manipulation (*n* = 40)	Simulated spinal manipulation (*n* = 40)
Participant characteristics		
Age (years)	32 (10)	38 (11)
Sex, Female	25 (63%)	25 (63%)
Marital status		
Single	19 (48%)	12 (30%)
Married	20 (50%)	27 (68%)
Divorced	1 (2.5%)	0 (0.0%)
Widowed	0 (0.0%)	1 (2.5%)
Weight (Kg)	75 (19)	81 (13)
Height (m)	1.8 (1.0)	1.7 (0.9)
Body Mass Index (Kg/m²)	26.1 (5.9)	28.8 (4.7)
Time of pain (months)	63 (69)	74 (78)
painDETECT score	7.5 (4.3)	9.3 (4.3)

Note: Data are presents as mean and standard deviation (SD) for continuous variables, and absolute values and proportions (%) for categorical variables

Twenty-seven of the 40 participants allocated to active treatment believed they had received active treatment, and 13 believed they had received placebo treatment. Similarly, 33 of the 40 participants in the placebo group believed they had received active treatment and seven that they had received a placebo (Chi-square test, *P* = 0.123). In the active treatment group, 36 reported expecting to feel ‘a little’ or ‘much’ better after treatment and in the placebo group, 37 had similar expectations (Chi-square test *P* = 0.299). There were no adverse events associated with the interventions.

### Primary outcomes

The spinal manipulation group had higher mean PPTs values in all body sites at post- intervention assessment. Sham group improved the PPT in 6 out of 12 body sites post-intervention assessment. The between-group mean difference in PPT favours the manipulation group at six of the twelve PPT sites (right L5, left L5, right L1, left L1, left DT, and right LE). Neither intervention altered postural stability. The crude differences between group means and their statistical significance are presented in Table 2.

**Table 2:** Pre- and post-intervention values of pain pressure threshold, postural stability and self-reported pain intensity for the spinal manipulation and simulated spinal manipulation groups

	Spinal Manipulation			Simulated Spinal Manipulation			Crude between-group mean difference (95%CI)		ANOVA *P* value	Adjusted between-group mean difference (95%CI)	ANCOVA *P* value
	Pre	Post		Pre	Post						
**PPT (Kgf)**											
Right MG	3.6 (1.3)	3.9 (1.5)		3.3 (1.0)	3.4 (1.0)		0.26 (-0.12 to 0.63)		0.175	0.20 (-0.03 to 0.77)	0.069
Left MG	3.7 (1.4)	4.0 (1.6)		3.4 (1.2)	3.5 (1.2)		0.26 (-0.06 to 0.57)		0.109	0.30 (-0.03 to 0.64)	0.077
Right AT	4.2 (1.3)	4.4 (1.5)		3.9 (1.2)	3.7 (1.5)		0.40 (-0.03 to 0.83)		0.064	0.40 (-0.06 to 0.87)	0.087
Left AT	4.3 (1.5)	4.6 (1.7)		3.8 (1.3)	3.7 (1.4)		0.34 (-0.12 to 0.80)		0.148	0.35 (-0.16 to 0.85)	0.174
Right L5	3.9 (1.7)	4.7 (2.3)		3.3 (1.4)	3.5 (1.5)		**0.55 (0.19 to 0.90)**		**0.003**	**0.47 (0.09 to 0.85)**	**0.017**
Left L5	3.8 (1.7)	4.5 (2.0)		3.3 (1.4)	3.6 (1.4)		**0.45 (0.13 to 0.76)**		**0.006**	**0.35 (0.01 to 0.68)**	**0.045**
Right L1	4.0 (1.7)	4.5 (1.9)		3.5 (1.3)	3.6 (1.5)		**0.41 (0.05 to 0.78)**		**0.026**	0.31 (-0.08 to 0.69)	0.117
Left L1	4.0 (1.8)	4.6 (2.0)		3.6 (1.4)	3.7 (1.4)		**0.57 (0.15 to 0.99)**		**0.008**	**0.45 (0.01 to 0.90)**	**0.049**
Right DT	3.3 (1.6)	3.5 (1.4)		3.0 (1.1)	3.0 (1.0)		0.16 (-0.17 to 0.50)		0.334	0.23 (-0.13 to 0.59)	0.200
Left DT	2.9 (1.1)	3.2 (1.2)		2.7 (1.1)	2.7 (1.0)		**0.35 (0.04 to 0.65)**		**0.028**	**0.38 (0.05 to 0.71)**	**0.026**
Right LE	2.7 (1.3)	3.0 (1.4)		2.4 (0.8)	2.3 (0.70)		**0.34 (0.08 to 0.60)**		**0.012**	**0.36 (0.08 to 0.64)**	**0.013**
Left LE	2.6 (1.0)	2.6 (1.0)		2.4 (0.7)	2.2 (0.6)		0.20 (-0.05 to 0.45)		0.120	0.18 (-0.09 to 0.46)	0.191
**CoP (cm²)**	51 (21)	54 (22)		60 (44)	54 (24)		9.2 (-5.4 to 23.7)		0.212	10.95 (-4.21 to 26.11)	0.154
**NPRS score**	5.1 (1.5)	2.3 (1.8)		5.5 (1.7)	3.5 (2.4)		-0.80 (-1.6 to 0.01)		0.054	-0.76 (-1.6 to 0.13)	0.093

Note: Values are expressed as mean and standard deviation (SD) for pre- and post-intervention. Significant differences between groups were tested using one-way analysis of variance (ANOVA) for crude between-group mean difference and one-way analysis of covariance (ANCOVA) for adjusted between-group mean difference with age, body mass index and participant beliefs about which treatment was received as covariates. Bold numbers represent statistically significant difference. Abbreviation: PPT - pressure pain threshold; Kgf - kilogram-force; MG - medial gastrocnemius muscle; AT - anterior tibial muscle; L5 - L5 vertebra; L1 – L1 vertebra; DT - deltoid muscle; LE - lateral epicondyle; CoP – Centre of Pressure; NPRS – Numeric Pain Rating Scale

### Secondary outcomes

There was no statistically significant difference in change in pain intensity between groups, though both groups experienced a statistically significant decrease in pain intensity after the intervention. Five body sites, namely right L5 [F(1,75) = 5.986, *p* = 0.017], left L5 [F(1,75) = 4.173, *p* = 0.045], left L1 [F(1,75) = 3.973, *p* = 0.049], left DT [F(1,75) = 5.162, *p* = 0.026], and right LE [F(1,75) = 6.504, *p* = 0.013] presented a greater increases in PPT scores for the manipulation group in the between-group comparison, after controlling for participant’s perceptions of the treatment, age and body mass index. One way ANCOVA revealed similar effects of the intervention on postural stability [F(1,75) = 2.071, *p* = 0.154] and pain intensity [F(1,75) = 2.891, *p* = 0.093], controlling for participant’s perceptions of the treatment, age and body mass index. The crude and adjusted estimates of between-group differences are depicted in Table 2.

**Fig. 4:**
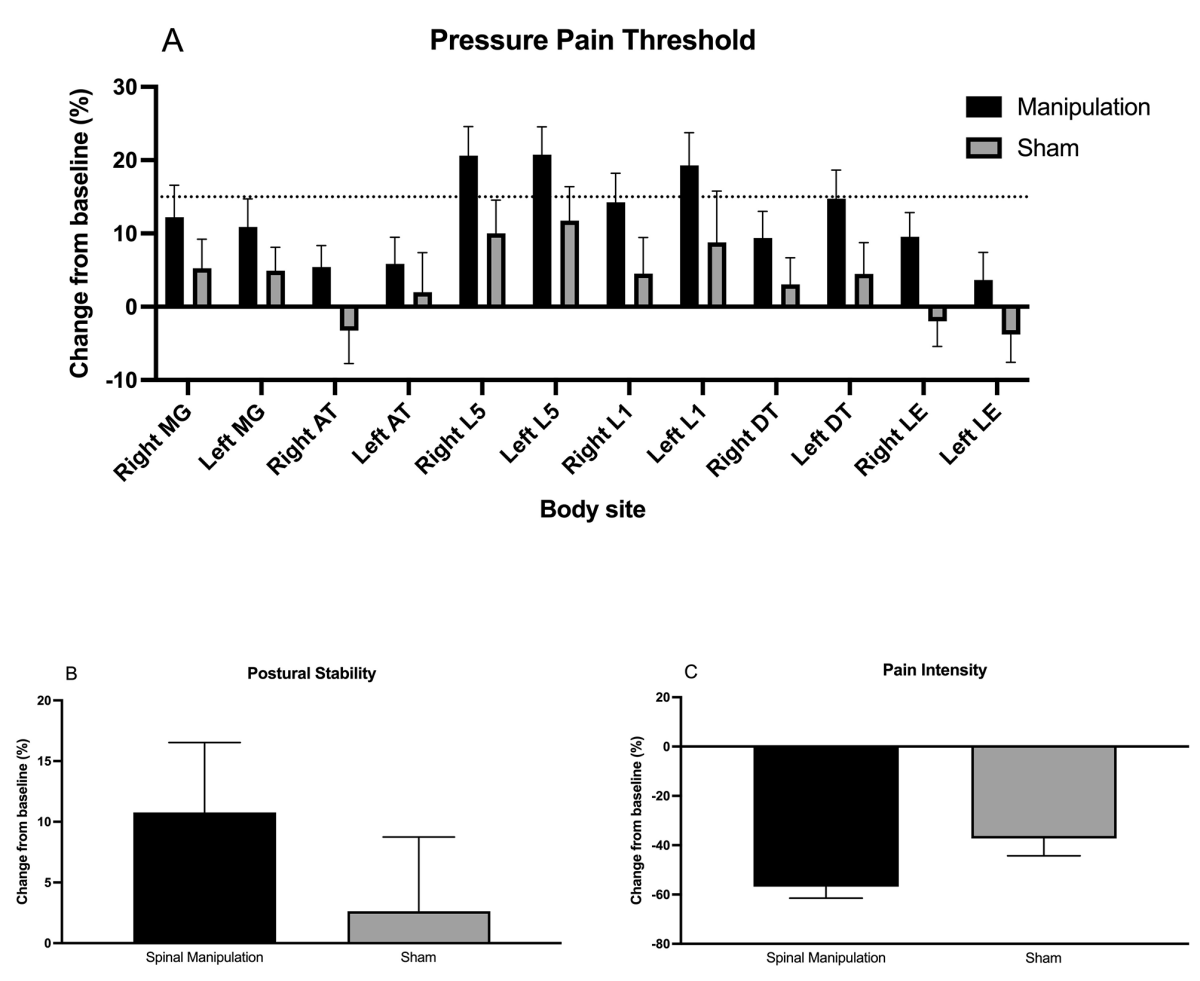
Change in pressure pain threshold (Panel **A**), postural stability (Panel **B**), and pain intensity (Panel **C**) percentages from post-intervention to baseline

Three lumbar body sites (right L5, left L5, and left L1) showed improvements in pressure pain threshold greater than 15% in the spinal manipulation group relative to the baseline measurements. The spinal manipulation group had a postural control variation of 11%, whereas the sham group had a variation of 3%. The self-reported pain intensity showed a decrease of 57% following the spinal manipulation and 37% after the sham intervention. Thirty-six (90%) of the participants allocated to active treatment attained minimal relevant clinical difference in pain intensity compared to 24 (60%) of the participants in the placebo group (Fisher`s exact test, *P* = 0.004). Fig. 4 shows the change in pressure pain threshold (Panel A), postural stability (Panel B), and pain intensity (Panel C) percentages from post-intervention to baseline.

### Compliance with the trial protocol

The interventions were applied as described in the registered protocol. All the documented outcomes were reported, but we changed the contextual factor analysis from the participant’s expectations regarding the treatment to the participant’s beliefs about the treatment received because the vast majority of participants had positive expectations of the treatments regardless of the group allocation. Besides, we calculated the percentage change from baseline adjusted for baseline values of the three continuous outcomes (pressure pain threshold, postural stability and pain intensity) for ease of interpretation.

## Discussion

This trial investigated the immediate effects of spinal manipulation on PPT, postural stability, and pain intensity in participants with cLBP. One session of lumbar spinal manipulation resulted in a local but not remote reduction in pain sensitivity compared to those who received a sham procedure. The magnitude of the treatment effect on lumbar pain sensitivity was small despite the significant between-group differences. Moreover, no substantial change in postural stability was observed between the two groups. Self-reported pain intensity showed clinically significant decreases in both groups after the intervention. Still, a higher proportion of participants in the spinal manipulation group achieved more than two points of pain relief. The participant’s belief in receiving the manipulation did not appear to have influenced the outcomes since the adjusted model revealed similar findings. Thus, spinal manipulation led to local pain relief, and this response is partly explained by contextual effects other than patient expectations, like patient-provider interactions or the treatment environment.

The reduction in lumbar pain sensitivity after the spinal manipulation presented here is not aligned with a recent review suggesting low-quality evidence of no difference in PPT after spinal manipulation compared to sham in musculoskeletal pain [39]. Another recent systematic review found no immediate hypoalgesic effect in patients with chronic pain after spinal manipulation and mobilisation with low certainty of evidence [40]. Thus, the reviews highlight that the true effect may differ substantially from the estimate due to several limitations of the published studies. For instance, spinal manipulation did not result in PPT changes compared with sham in a sample of patients with and without LBP at the time of the intervention [17]. The inclusion of pain-free participants may have contributed to underestimating the lumbar hypoalgesic effects after lumbar manipulation.

Our findings suggest that spinal manipulation likely led to a physiological hypoalgesic effect in the lumbar spine rather than other nonspecific effects. Prior research identified that the enhancement in lumbar PPT after spinal manipulation occurs through a neurophysiological effect affecting the entire lumbar spine [15, 41]. The authors also observed an increase in lumbar PPT in cases with no clinical pain relief [15]. On the other hand, our findings revealed a similar hypoalgesic response to spinal manipulation and the sham intervention at remote sites, corroborating prior studies in musculoskeletal pain [39]. Although we found improvements in pain sensitivity at two out of eight remote sites, there is an absence of biological plausibility for these findings in only two random remote regions. Thus, lumbar spinal manipulation may be effective at increasing the PPT solely in the lumbar region due to the acute small pain relief over a sham procedure for patients with cLBP.

Prior studies argue that LBP impairs balance [19–21], which leads to the hypothesis that interventions that ease the pain could improve postural stability. In this trial, we found no significant changes in postural stability post-intervention in either group, corroborating a previous study [16]. Immediate post-treatment examination may not have revealed changes in postural stability, as pain relief may take longer to induce neuromuscular changes that would interfere with balance. Alternatively, clinicians and researchers may accept that spinal manipulation is likely ineffective at improving postural stability in patients with cLBP.

Contextual factors and therapeutic touch are known to have clinical relevance for musculoskeletal pain [23]. In our study, most participants in both groups had positive expectations about treatment and clinically significant pain relief in the patient-reported outcome measure. The interventions improved subjective lumbar pain relief (spinal manipulation = 57% and sham = 37%) more than semi-objective outcomes (lumbar PPT: spinal manipulation nearly 20% and sham almost 10%). Placebo interventions can exert a relevant influence on subjective outcomes [42]. The low number of participants recruited for the current study was insufficient to reach statistical significance for subjective pain relief in between-group comparison, which was a secondary study aim. Indeed, our study was planned to detect a minimum difference of 15% (effect size of 0.64) in the lumbar PPT, which was observed in the lumbar PPTs of the spinal manipulation group. Arguably, spinal manipulation provided a marginal effect on pain relief compared to a sham treatment. A future study with a large sample size likely enhances the precision of the estimate. The reduction in pain observed in the sham group can be explained by the analgesic effect of the touch-based approach, previously reported in individuals with LBP [43], or by contextual effects other than patient expectations.

This research has relevant clinical implications. The current study extended the body of knowledge on pain relief after spinal manipulation. In patients with cLBP treated with a spinal manipulation session, we identified a modest treatment benefit in local pain sensitivity superior to a placebo intervention. Placebo intervention has a trivial effect on pain intensity compared to no intervention in patients with cLBP [26] and other musculoskeletal conditions [42]. In the current study, spinal manipulation provided pain relief in conjunction with the placebo effect in an additive manner since we observed greater improvement in the active group relative to baseline. We designed a clinical trial to measure the specific effect of spinal manipulation in patients with cLBP, controlling for nonspecific effects and some contextual factors like patient expectations. Our finding is particularly important since patients with LBP consider immediate relief an acceptable treatment outcome [44]. Both groups achieved an average of more than subjective 20% pain relief, the smallest worthwhile effect estimated for patients with cLBP treated with physiotherapy [45], though more participants in the intervention group reached this minimum clinically important change. Spine manipulation is an easily accessible and safe method for treating patients with cLBP. Therefore, lumbar spinal manipulation must be used in patients with cLPB to promote immediate pain relief.

### Strengths and limitations

Objective measures are rarely used in clinical trials involving simulated interventions and can potentially inform an intervention’s specific contribution. In addition, we investigated the analgesic effect of the lumbar spinal manipulation at the participant complaint site and in remote areas to assess the potential systemic response to the intervention. Although the current study emphasises pain sensitivity and postural instability, we examined self-reported pain intensity, the major subjective measure for LBP [46]. Lastly, the experimental procedure successfully blinded the participants, balancing context-dependent effects.

Nonetheless, our study has many methodological weaknesses. For instance, PPT is a semi-objective measure and may be influenced by contextual factors involved in the therapeutic encounters. The reliability of the baropodometres in measuring postural stability needs to be established, although baropodometres showed concurrent validity compared to the force plate. Besides, the impossibility of blinding the therapist to the intervention due to the nature of the interventions and centre-specific effects may have influenced the findings since the participants were recruited from a single centre. Furthermore, the absence of a follow-up period represents a shortcoming of the current study. Additional time points evaluating pain sensitivity would be helpful for identifying the wash-out period of the spinal manipulation. Ultimately, analysing participants’ perceived treatment allocation is likely underpowered and may be useful for generating hypotheses solely. Likewise, the widths of the confidence intervals were adjusted for covariates but not for a multiplicity of inferences, which may impact the study findings.

## Conclusion

One spinal manipulation session reduces lumbar pain sensitivity but does not affect postural stability compared to a sham session in individuals with cLBP. Remote pain sensitivity remained unchanged for both groups. After the intervention, there was a marked decrease in self-reported pain intensity in both groups, and a higher proportion of participants in the spinal manipulation group reached clinically significant pain relief. The participant’s belief in receiving the manipulation did not appear to have influenced the outcomes since the adjusted model revealed similar findings.

## Data Availability

The datasets used during the current study are available from the corresponding author on reasonable request.

## Declarations

### Trial registration

The trial was registered at the Brazilian Registry of Clinical Trials – RBR-3ksq2c (WHO U1111-1252-3077).

## Abbreviations

ANOVA: Analysis of Variance
BMI: Body Mass Index
cLBP: chronic Low Back Pain
CONSORT: Consolidated Standards of Reporting Trials
CoP: Centre of Pressure
LBP: Low Back Pain
NPRS: Numeric Pain Rating Scale
REBEC: Brazilian Registry of Clinical Trials
SD: Standard Deviation
SPIRIT: Standard Protocol Items: Recommendations for Interventional Trials
TIDieR: Template for Intervention Description and Replication
UNICENTRO: Midwestern Parana State University
UNIGUAIRACA: Guairacá University Centre
